# Supplementary material for: Spike-based alignment learning solves the weight transport problem
Source: Nat Commun. 2026 Jul 7;17:8699. doi: 10.1038/s41467-026-74460-8 (PMC13490435; doi:10.1038/s41467-026-74460-8)
Supplement: Supplementary file 1 [file 41467_2026_74460_MOESM1_ESM.pdf]

Spike-based alignment learning solves the weight transport problem

Supplementary Information

Supplementary Note 1: Analysis of the Kolen-Pollack weight decay rate in spiking sampling networks

In the following, we access the influence of the weight decay rate λ on both the symmetrization and the Kullback-Leibler divergence (D_{KL}) in spiking sampling networks (SSNs) trained with the Kolen-Pollack algorithm (KP) (Eqn. 5). While a higher λ results in better symmetrization, it also interferes with the wake-sleep algorithm itself: As performance improves and the weight updates from wake-sleep reach the same magnitude as the weight decay, learning will stall. Hence, the goal of achieving low D_{KL}s demands small decay rates, giving rise to an inevitable performance trade-off between gradient descent and weight symmetrization updates as demonstrated in Supplementary Fig. 1 (left column).

Moreover, given a finite training time, each noise level has a different optimal λ for achieving the lowest D_{KL} (compare the minimum for the orange and blue curves in the D_{KL} of the synaptic noise scenario). This would effectively require adapting the weight decay rate to the – usually unknown – noise levels of the system. For the synaptic noise scenario in Supplementary Fig. 4, we choose the λ corresponding to the minimum of the $\sigma_{\mathrm{syn}}^{\mathrm{noise}} = 0.8$ curve.

The presence of plasticity noise poses another issue: The standard Kolen-Pollack algorithm (KP) algorithm uses identical decay rates on both synapses, which are fundamentally incapable of counterbalancing the asymmetric weight updates originating from noisy plasticity. Hence, weight decay has little effect on the symmetry, unless the decay rate is so strong that it will suppress the wake-sleep learning (Supplementary Fig. 1, right column). For the plasticity noise scenario in Supplementary Fig. 4, we choose a λ of 4×10^{-5}, which doesn't deteriorate the wake-sleep learning too much but still has a measurable effect on the weight symmetry.

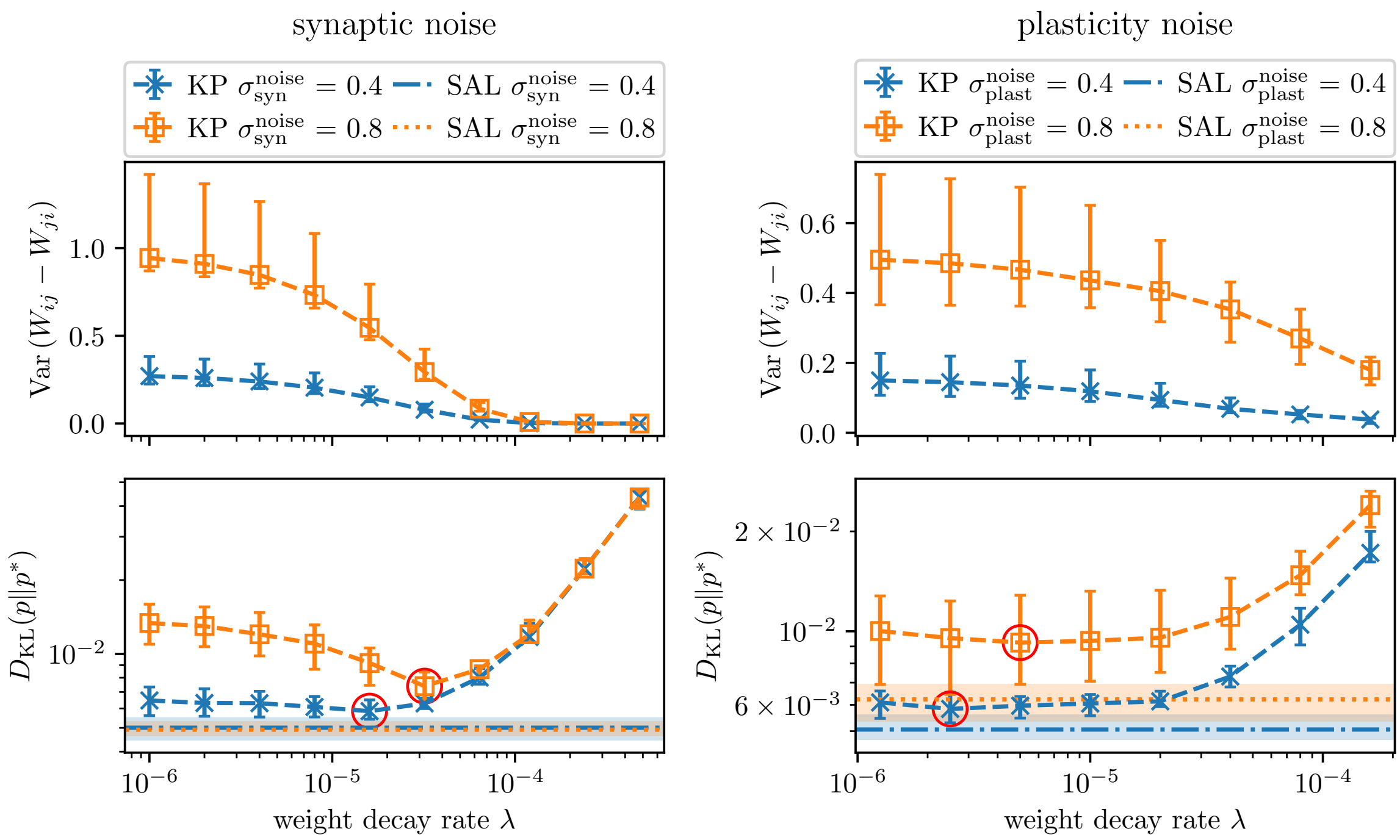

Supplementary Figure 1: Choosing the optimal Kolen-Pollack weight decay rate λ for the SSN experiment. Depicted is the final asymmetry and D_{KL} after 2000 wake-sleep cycles as a function of the weight decay rate λ. The straight lines are the bottom of the lower panels show the spike-based alignment learning (SAL) results for reference. The lowest D_{KL} values are encircled in red.

Supplementary Note 2: Validation of the analytical computation of spike-timing difference distributions

Supplementary Fig. 2 demonstrates that the spike-timing difference distributions (STDDs) computed with the analytical method (Eqns. 14 to 29) agrees with a numerical simulation of an equivalent two neuron system. For each panel, weights and biases are randomly drawn from a uniform distribution $\mathcal{U}(-1, 1)$. The time constant of the alpha-shaped PSP is chosen to be $\tau_{\mathrm{syn}} = \frac{1}{3}\tau_{\mathrm{ref}}$, the total simulation time is 33 000 τ_{ref}. All four panels show good agreement between the analytical method and the simulation.

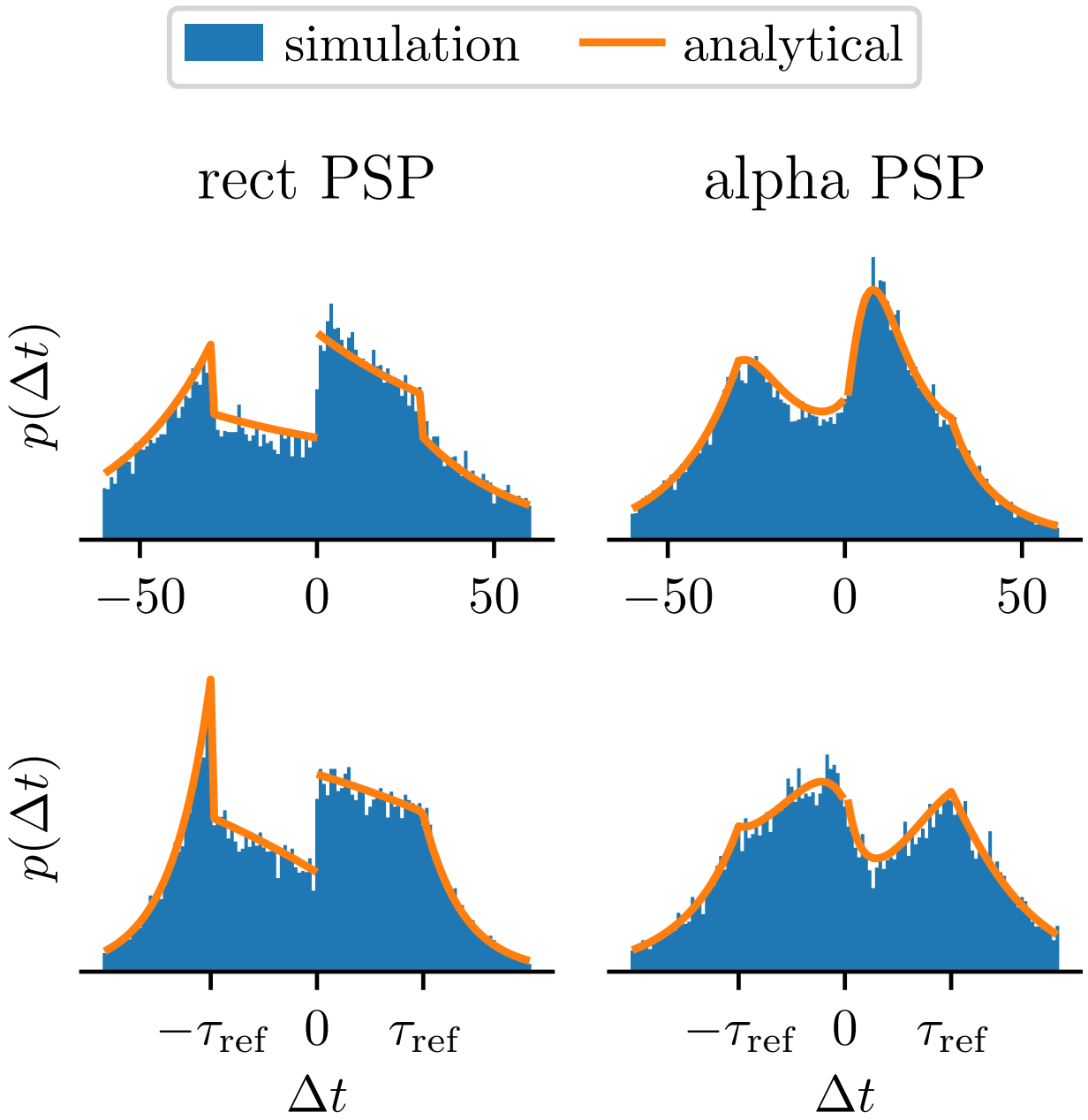

Supplementary Figure 2: Comparison between the analytical method to compute STDDs and numerical simulations.

Supplementary Note 3: Visualization of the STDD deviation variables $\delta(t)$ and $\Delta(t)$

For illustrative purposes, we show in Supplementary Fig. 3 two exemplary curves of the STDD deviation variable $\delta(t)$ and the integrated deviation $\Delta(t)$ as defined in Eqn. 30. The curves are computed using the analytical method presented in Section for a two-neuron system with rectangular post-synaptic potentials (PSPs) with two excitatory connections ($W_{ij} = Wji = 0.5$) and two inhibitory weights ($W_{ij} = Wji = -0.5$), unequal biases ($b_i = 0$, $b_j = -1$) and a deviation of $\varepsilon = 0.05$.

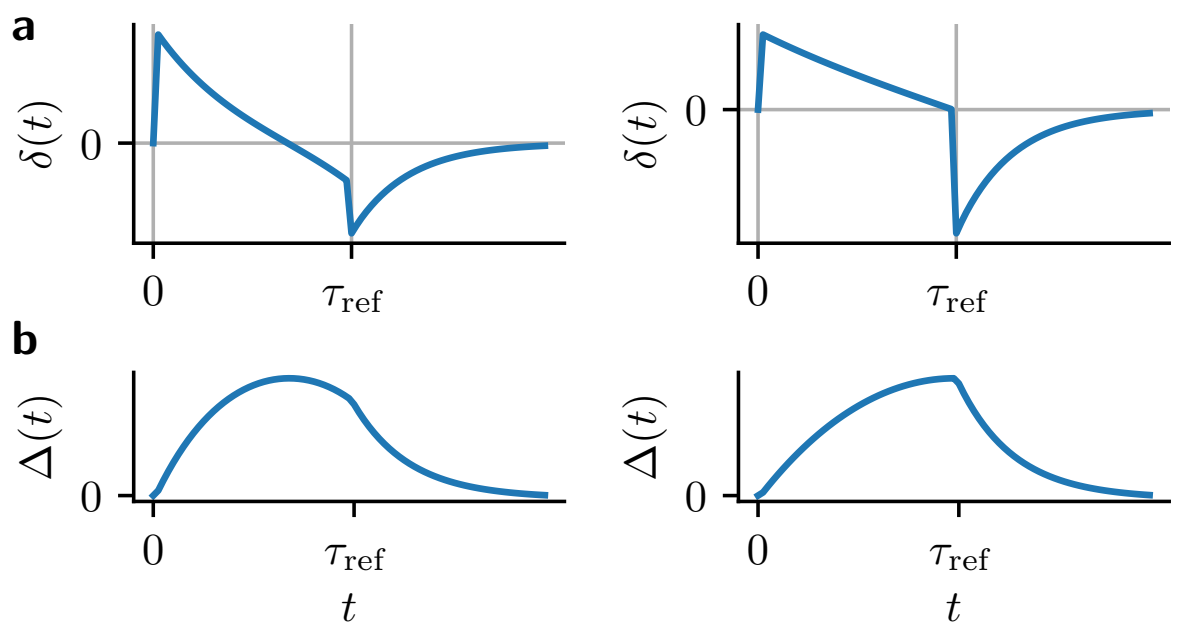

Supplementary Figure 3: Illustration of the STDD deviation variables $\delta(t)$ and $\Delta(t)$. a STDD deviation $\delta(t)$ for $W_{ij} = W_{ji} + \varepsilon$ (with $\varepsilon = 0.05$) and rectangular PSPs, for positive (left) and negative weights (right). **b** Integrated deviation $\Delta(t)$ for positive (left) and negative (right) weights.

Supplementary Note 4: Extended comparison of symmetrization algorithms in deep networks

For further contextualizing the results of the SymmNet experiment presented in Supplementary Fig. 7, we additionally train the ConvNet with two more symmetrization algorithms:

1. A combination of backpropagation (BP) in the fully connected layers and feedback alignment in the convolutional layers (BP + FA). Due to the weight sharing problem in the convolutional layers, any symmetrization algorithm is only applied to the fully connected layers only in the SymmNet architecture. Using BP in the fully connecting layers models the best achievable symmetrization (namely when $\boldsymbol{B} = \boldsymbol{W}^\intercal$ is enforced all the time) and therefore serves as a fair upper limit for any real symmetrization scheme.

2. One variant of sign-concordant feedback alignment (SCFA) given by $\boldsymbol{B}_t = (\boldsymbol{W}_t)|\boldsymbol{B}_0|$, where $\boldsymbol{B}_0$ is a fixed random backward matrix [1]. However, this implementation still requires to transport the sign of the forward weight.

The results are shown in Supplementary Table 1. Among all symmetrization algorithms, SAL comes closest to the optimal limit given by BP + FA due to its ability to best align forward and backward weights.

Supplementary Table 1: Final test accuracies of the SymmNet experiment, augmented by a combination of BP in the fully connected layers and feedback alignment (FA) in the convolutional layers (BP & FA) as a model for the best possible symmetrization algorithm in this setting and sign-concordant feedback alignment (SCFA) algorithm.

	BP	BP + FA	FA	SC FA	SAL	KP	RDD
CIFAR-10	83.8±0.3	80.4±0.3	69.5±0.7	78.4±0.5	79.6±0.4	78.7±1.0	78.7±0.4
FMNIST	92.1±0.1	91.5±0.3	90.0±0.1	91.4±0.2	91.5±0.2	90.8±0.3	91.5±0.1
SVHN	93.5±0.1	91.9±0.1	84.0±0.5	90.5±0.2	91.5±0.1	90.9±0.1	89.2±0.3

Supplementary Tables

In the following, we provide the relevant simulation parameters for all numerical experiments.

Supplementary Table 2: Simulation parameters for the SSN experiments (Fig. 4).

	synaptic noise			plasticity noise		
	w/o SAL	w/ SAL	KP	w/o SAL	w/ SAL	KP
$\boldsymbol{W}^*$	$\mathcal{U}[-1,1]$					
$\boldsymbol{b}^*$	$\mathcal{U}[-1,1]$					
$\boldsymbol{W}_{\text{init}}$	$\mathcal{N}(0,0.2)$					
$\boldsymbol{b}_{\text{init}}$	$\mathcal{N}(0,0.2)$					
$\sigma^{\text{noise}}_{\text{syn}}$	variable	variable	variable	0.2	0.2	0.2
$\sigma^{\text{noise}}_{\text{plast}}$	0	0	0	variable	variable	variable
$\eta_{\boldsymbol{W}}$	0.01	0.01	0.01	0.001	0.001	0.001
$\eta_{\boldsymbol{b}}$	0.01	0.01	0.01	0.001	0.001	0.001
η_{SAL}	0	0.01	0	0	0.002	0
λ	0	0	4×10^{-5}	0	0	4×10^{-5}
simulation time step	0.2 ms					
τ_{ref}	10 ms					
duration sleep phase	10 s					

Supplementary Table 3: Simulation parameters for the cortical microcircuits (Fig. 5).

	BP	FA	SAL
λ^{api}		0.02	
λ^{nudge}		0.6	
λ^{bas}		1.0	
λ^{den}		1.0	
simulation time step		1 ms	
$t_{\text{moving average}}$		2 s	
τ_{ref}		10 ms	
η for $W^{\text{PP}}_{1,0}$	0.2	0.2	0.2
η for $W^{\text{PP}}_{2,1}$	0.003	0.003	0.003
η for $B^{\text{PP}}_{1,2}$	n.a.	0.0	0.001
b_1		-1.0	
b_2		-1.0	
teacher $W^{\text{PP}}_{1,0}$		2.0	
teacher $W^{\text{PP}}_{2,1}$		2.0	
training values per iteration		20	
validation valuesper iteration		6	
values for SAL per iteration	0	0	5
presentation time		20 s	
student init $W^{\text{PP}}_{1,0}$		$\mathcal{U}[-3,3]$	
student init $W^{\text{PP}}_{2,1}$		$\mathcal{U}[-3,3]$	
student init $B^{\text{PP}}_{1,2}$	$=W^{\text{PP}}_{2,1}$	$\mathcal{U}[-3,3]$	$\mathcal{U}[-3,3]$

Supplementary Table 4: Simulation parameters for the preliminary two layer study (Fig. 6).

	Common Settings
input size	50
output size	50
$\boldsymbol{W}_{\text{init}}$	uniform Kaiming He [2]
$\boldsymbol{B}_{\text{init}}$	uniform Kaiming He
	SAL
simulation time step	1 ms
learning rate	0.05
τ_{ref}	10 ms
	RDD[a]
stimulation ratio	0.2
RDD time	1 s
	STDWI
simulation time step	0.25 ms
stimulation ratio	0.2
stimulation correlation	0.0
stimulation rate	200 Hz
stimulation duration	100 ms
stimulation weight scale	10
weight scale	75
STDP τ_{fast}	10 ms
STDP τ_{slow}	200 ms
learning rate	0.02
decay weighting	0.1

[a] All regression discontinuity design (RDD)-specific model parameters are the same as in the original publication [3].

Supplementary Table 5: Simulation details for the SymmNet experiment (Fig. 7).

Symbol	Parameter name	CIFAR-10	SVHN	Fashion-MNIST
		ConvNet architecture		
	input size	$32 \times 32 \times 3$		$28 \times 28 \times 1$
	conv layer 1	Conv2D $(5 \times 5) \times 64$, ReLU<br>MaxPool2d (kernel size = 2, stride = 2)		
	conv layer 2	Conv2D $(5 \times 5) \times 64$, ReLU<br>MaxPool2d (kernel size = 2, stride = 2)		
	FC layer 1	size 384, ReLU		
	FC layer 2	size 192, ReLU		
	FC layer 3	size 10, softmax		
		ConvNet parameters		
$\boldsymbol{W}_{\text{init}}$, $\boldsymbol{b}_{\text{init}}$	initialization	uniform Kaiming He[2]		
	batchsize	64		
$\mathcal{L}$	loss function	cross-entropy		
	optimizer	stochastic gradient descent		
$\eta_{\boldsymbol{W},\boldsymbol{b}}$	learning rate	0.01		
μ	momentum	0.9		
		symmetrization parameters		
$\boldsymbol{B}_{\text{init}}$	initialization	uniform Kaiming He[a]		
		KP[b]		
λ	weight decay factor	0.001		
		SALNet[b]		
	layer sizes	1600, 384, 192 and 10		1024, 384, 192 and 10
	neuron model	GLM with rectangular PSP		
simulation time step	1 ms			
τ_{ref}	refractory period	10 ms		
τ_{syn}	synaptic time constant	10 ms		
η_{SAL}	SAL learning rate	0.04		
	number of updates per epoch	5		
	duration per update	64 s		
		RDDNet[bc]		
	layer sizes	1600, 384, 192 and 10		1024, 384, 192 and 10
	neuron model	LIF with RDD stimulation protocol		
T	total stimulation time	90 s		

[a] Not applicable to BP, because there are no explicit backward weights.
[b] Only applied to fully-connected (FC) layers 1 – 3. The convolutional layers are trained with FA.
[c] All RDD-specific model parameters are the same as in the original publication [3].

Supplementary Table 6: Simulation parameters for the Dale's law compatible two neuron system (Fig. 9).

	Network obeying Dale's law
$W^{EE}_{12,\,21}$	can vary between 0 and 2
$W^{IE}_{11,\,22}$	$\sim \mathcal{N}(4, 0.2)$
$W^{IE}_{12,\,21}$	$\sim \mathcal{N}(-1, 0.2)$
$b^{E}_{1,\,2}$	$\sim \mathcal{N}(0, 0.2)$
$b^{I}_{1,\,2}$	$\sim \mathcal{N}(-2, 0.2)$
PSP kernel	rectangular with $\tau_{\text{syn}} = \tau_{\text{ref}}$

Supplementary References

1. Liao, Q., Leibo, J. & Poggio, T. *How important is weight symmetry in backpropagation?* in *Proceedings of the AAAI Conference on Artificial Intelligence* **30** (2016).
2. He, K., Zhang, X., Ren, S. & Sun, J. *Deep Residual Learning for Image Recognition* in *2016 IEEE Conference on Computer Vision and Pattern Recognition (CVPR)* (IEEE, Las Vegas, NV, USA, June 2016), 770–778. ISBN: 978-1-4673-8851-1.
3. Guerguiev, J., Kording, K. P. & Richards, B. A. *Spike-based causal inference for weight alignment* 2019. arXiv: 1910.01689 [q-bio.NC]. https://arxiv.org/abs/1910.01689.
