# Supplementary material for: Spike-based alignment learning solves the weight transport problem
Source: Nat Commun. 2026 Jul 7;17:8699. doi: 10.1038/s41467-026-74460-8 (PMC13490435; doi:10.1038/s41467-026-74460-8)
Supplement: Supplementary file 2 [file 41467_2026_74460_MOESM2_ESM.pdf]

nature portfolio

Peer Review File

Spike-based alignment learning solves the weight transport problem

Corresponding Author: Mr Timo Gierlich

This file contains all reviewer reports in order by version, followed by all author rebuttals in order by version.

Version 0:

Reviewer comments:

Reviewer #1

(Remarks to the Author)
Summary: The authors propose a novel method for dynamically symmetrizing connectivity in stochastic spiking networks that learn either via backpropagation or the wake-sleep algorithm for Boltzmann machines. In contrast to Kolen-Pollack learning, which relies on weight decay to symmetrize connectivity, the authors use an anti-causal STDP rule to establish weight symmetry as a line attractor in weight update dynamics (absent other learning signals), as a spiking analog to phaseless alignment learning.

Overall opinion: The algorithm (SAL) appears novel, and the mathematical proofs supporting it are interesting. The work will likely be of use to researchers in theoretical neuroscience and neuromorphic computing. I think that this paper is worth publishing with the addition of some empirical comparisons to alternative learning algorithms that highlight the algorithmic merit and unique biological implications of SAL.

Major criticisms:
1. In recent years, there has been a proliferation of methods that can achieve weight symmetry (or alternative types of learning without weight transport), to the point that one has very little idea of how to compare these algorithms. Many symmetrization methods use only local information, and many are capable of training neural networks on simple datasets (e.g. MNIST, CIFAR10). The authors do a fairly good job of reviewing these different algorithms in the intro and discussion, but do not provide any concrete empirical comparisons beyond showing superior learning in comparison to feedback alignment, which is by now well known to perform poorly on even relatively simple tasks. In particular, I think that the authors have been unfair to the Kolen-Pollack algorithm in several places, and that empirical/mathematical demonstrations are required to support their claims. They introduce a concept called the ‘weight update transport problem’ in both the introduction and discussion, which argues that the symmetric updates in the Kolen-Pollack algorithm are implausible. However, for Boltzmann machines for instance, the KP algorithm is incredibly simple, amounting to adding a constant weight decay term to the ordinary Hebbian-style update. An argument as to why this type of update is less plausible than the SAL algorithm requires much more explanation in my opinion. Furthermore, in terms of performance, I think that the experiments that the authors conduct are highly unlikely to reveal that SAL is an improvement over KP. This is not to say that SAL doesn’t have advantages–I just think that different experimental and/or mathematical demonstrations will likely be required. For instance, the use of a constant decay term in the KP algorithm could present serious issues for continual learning, which the SAL algorithm is unlikely to suffer from (because it establishes symmetric weights as a line attractor).

2. Related to the issue above, the authors do little to explain how SAL could be validated at an experimental level, relative to alternative symmetrization algorithms. SAL uses only ingredients that have been observed biologically (various types of STDP curves), but so does Kolen-Pollack, for instance, requiring in the case of Boltzmann machines essentially only Hebbian plasticity and gradually decaying synaptic strengths. The authors allude to SAL’s ability to better accommodate neurons with differing physiological properties, but do not provide comparisons to alternative symmetrization algorithms. This is a missed opportunity in my opinion–for instance, in neuron models with multiple synaptic connections from neuron A to neuron B, KP and SAL may differ in their predictions for how these multiple synapses should jointly evolve throughout time, and may provide a method to experimentally differentiate between the two algorithms. Providing empirical evidence that SAL is better able to accommodate neural networks with E-I separation would also be beneficial, as this is a claim that the authors make textually but do not empirically substantiate.

3. Lastly, the authors do not adequately compare SAL to algorithms that do not require feedback symmetry. They cite and briefly discuss pseudo-inverse-based learning algorithms like difference target propagation and the wake-sleep algorithm for Helmholtz machines, but do not provide any actual comparisons: this is particularly problematic, because the authors do not justify the biological plausibility of symmetric connectivity in the first place. To my knowledge, symmetric connectivity is only justifiable at the level of a very coarse approximation between brain areas, and certainly cannot hold between excitatory and inhibitory neurons (because it violates Dale's law, as the authors themselves discuss). If the weight transport problem can be solved by regression from higher-order cortical areas to lower cortical areas, why should we be worrying about symmetry in feedback connections? In contrast to the KP algorithm, I do not think that empirical comparisons are required here: I would rather see a discussion of the experimental evidence supporting these different families of models.

Minor criticisms:
1. Line 90: the authors cite the Helmholtz machine 'wake-sleep' learning algorithm, but I believe that they intend to cite the original Boltzmann machine paper (or possibly the contrastive divergence learning algorithm), as this is the method that they actually use. People sometimes refer to the contrastive divergence learning algorithm as a 'wake-sleep' learning algorithm, which is semantically true but liable to cause confusion. The Wake-Sleep learning algorithm for Helmholtz machines does not require feedforward and feedback weight symmetry, which could make a mistaken citation particularly confusing in this context.

2.Line 748-9: the claim that mirrored STDP cannot equilibrate initial weight differences is confusing. If it is a variant of the Kolen-Pollack algorithm, which does cause weight symmetry, why would mirrored STDP fail to do this?

3. Line 999: I do not think that $W_{12} = W_{21}$ is a unique fixed point. The entire argument is that this equality applies to an entire continuous line attractor, not a unique fixed point.

4. Does the proof in section 4.2.2 apply equally well to a full recurrent neural network, or is it fundamentally restricted to the 2 neuron case?

5. The authors claim to provide the first spiking network implementation of the learning algorithm described in the Sacramento et al. 2018 paper. It would be good to cite and discuss the Guerguiev et al. 2017 paper, "Towards deep learning with segregated dendrites", which does use spiking neural networks in conjunction with a bio-plausible error propagation algorithm.

(Remarks on code availability)

Reviewer #2

(Remarks to the Author)
Summary
This paper presents a mechanism called spiked-based alignment learning (SAL), allowing to align/symmetrize reciprocal weights based on a particular form of spike-timing dependent plasticity (STDP) arising from the spike-timing difference distribution (STDD) between two neurons. It relies on the presence of noise in neuronal systems to get this STDD information. This paper demonstrates the functionality of SAL in two use cases: (i) a spiking sampling network (SSN) relying on a wake-sleep training and (ii) a dendritic cortical microcircuit learning a nonlinear input-output mapping, with a teacher network training a student one. In addition, this paper shows that SAL remains valid for different types of post-synaptic potentials (PSPs) – rectangular, alpha-shaped – and discusses the implications of different PSPs on the algorithm. One of the key claims of SAL is that is well-suited to analog hardware as it operates based on noise and is robust against components' mismatch.

Strengths and weaknesses
The main strength of this paper is the idea of using STDD information to enable the alignment of reciprocal weights in neuronal systems, subject to both noise and mismatch. Significant work is provided in demonstrating this mechanism based on simulation results in different conditions (two use cases, different PSPs, etc), and based on theoretical proofs at the end of the paper. This key claim is thus well-supported by the different experiments and results discussed in the current version of the paper.
The main weakness is that the use cases tackled with the proposed SAL technique remain at a relatively low level, in the sense that these are relatively simple problems, and it is not extremely clear to me how this will extend to more challenging problems. I would thus encourage the authors to clarify the computational overhead of SAL and the extent to which noise is necessary for it to work and impacts the learning process. In addition, results on at least one conventional spiking dataset would be of great value to the reader and would provide some intuition about how the proposed SAL technique can scale to more complex problems.

Major comments
• What is the computational overhead of SAL ? What would be the best way to implement it in an analog substrate ?
• One of the key assumptions for SAL to work is that noise is present in the considered system. Therefore, I am wondering how SAL would work in hardware implementations for which intrinsic noise is very limited, for example a digital hardware implementation ? Would SAL still work in that context, or does that depend on the level of noise in the input data ? In addition, is there a minimum level of noise necessary to ensure a proper operation ? How does the noise level impact the convergence speed of the weight alignment ?

• Would that make sense to use some alignment metric to assess the alignment of forward and backward weights, similar to the angle typically used in works related to feedback alignment (FA) ?
• The paper does a good job at demonstrating the operation of SAL but remains limited to some simple problems. I believe the paper would be strengthened if you could demonstrate the proper operation of SAL on at least one conventional yet simple spiking classification dataset, with alignment metrics at the level of whole matrices, if feasible in the time allowed by the review process

Minor comments
• p.4 Fig. 2: For Fig. 2c1, I would explicitly add that the top graph is for two excitatory weights and the bottom one for inhibitory weights. We can infer that from the weights values on the right side, but I still believe it would be clearer to write excitatory and inhibitory in plain text. For Fig. 2d, I would add a legend for the colors instead of just describing it in the caption. In addition, how does the convergence speed compare with tau_ref ? Is there a link between the convergence speed and tau_ref ?
• p.7 l.538: It should be Fig. 6b2 instead of Fig. 6a2, no ?
• p.8 Fig. 6b2: I would indicate with a vertical line the point at which the B_12 switches sign and thus provides a meaningful error signal
• p.9 Fig. 7b: I recommend to zoom on the STDD depicted here as it is difficult to see the differences due to the biases based on the current figure

Dr. Martin Lefebvre, TU Delft

(Remarks on code availability)

Reviewer #3

(Remarks to the Author)
This submitted work is an interesting approach which bridges between spiking (sampling) network modelling, addressing of the weight-transpose problem of credit assignment, and giving a purpose to noise within neural networks. In general it is extremely well written, the figures are clear and cover every desirable aspect of network analysis, and the theory is sound. The overall framing of the problem is also done very well and I enjoyed the perspective.

However, there are two major flaws at present which make it difficult to recommend for acceptance. One, a lack of acknowledgement and embedding of this work within a number of papers which already attempt weight alignment in deep and spiking neural networks via noise for parameter estimation (or update alignment). And two, the lack of a test of this method in training of more difficult tasks, task scales at which these methods really could be shown to break down.

Returning to the first concern, there is an unfortunate and worrying lack of embedding of this work within the larger literature on this topic. This work, as it is presented, misses out on key papers which have already covered a number of the topics that are attempted herein. Most importantly, two key papers have already considered how in spiking neural networks it might be possible to use spike timing (with or without membrane voltages) in order to estimate symmetric reciprocal weight values. The first, "Spike-based causal inference for weight alignment" by Guerguiev et al. (2019) describes how causal inference (via regression discontinuity design) can be used to directly infer the values of synaptic weights of a forward-driven model for use in a backward pass. Even more similarly to this work, "Overcoming the Weight Transport Problem via Spike-Timing-Dependent Weight Inference" by Ahmad et al. (2020) proposes that an STDP rule may be used with for weight inference that has a similar form to that proposed in this work. Namely an anti-hebbian form which can be shown to carry out a weight inference process of forward weights using spike-timing alone.

Aside from these two (extremely relevant) works which aught to be considered, a paper by Akrout et al. 2020 ("Deep Learning without Weight Transport") is not cited and is close to a rate-equivalent alternative to SAL (better to compare against that the Kolen-Pollack algorithm. Further, "Biologically-plausible learning algorithms can scale to large datasets" by Xiao et al. (2018) provides more large scale evidence for sign-alignment as a fruitful direction.

These concerns around embedding within the existing literature are significant from my perspective. Their exclusion (especially Guerguiev et al. and Ahmad et al.) means that this work is not compared against true contemporary and comparable research. The existence of these works also impacts the novelty of this approach.

My second major concern lies around the scale of tasks attempted by this work. Though I acknowledge that machine learning benchmarks are not the only important tasks, all serious breakdowns of training by FA/DFA/TP or other 'biologically-plausible' algorithms tend to breakdown only at a certain scale of task complexity (specifically close to ImageNet if one considers the work by Bartunov et al. 2018). Given this, I would be curious to see how well SAL truly does when applied in networks to attempting such tasks (though I also understand that this require a significant network change).

One final small concern is that it appears that Figure 5b1 shows that the divergence begins to grow near the end of training (as does the variance across models) for the SAL model. Is this indeed the case? Would these networks diverge in accuracy if allowed to train for longer? If so this should be a concern, so I'd like to see longer simulation time for these plots.

Overall, I think there are a number of aspects of this work that are truly interesting and novel, including the solving of weight-

transport within models with simultaneously active forward and backward weights, the clear theoretical relationship to spike timing differences, and the strong convergence properties and demonstrations. It is also extremely well written, formatted, and presented. However in it's current state this paper sidelines much of existing work that has been done in this direction and this should be rectified.

(Remarks on code availability)

Version 1:

Reviewer comments:

Reviewer #2

(Remarks to the Author)
I thank the authors for their work in addressing my comments.
With the added results on common machine learning datasets, as well as with the comparison to other conventional feedback alignment algorithms, I believe the paper is now ready for publication and do not have any further comments.

Dr. Martin Lefebvre, TU Delft

(Remarks on code availability)

Reviewer #3

(Remarks to the Author)
I want to start by commending the authors on significant improvements and integrations of this work with existing literature in the new revision. It is much improved and addresses almost all of my concerns.

The added comparison to RDD is a great addition and likely involved a lot of work. However, I find it odd that the authors chose not to also compare against STDWI? STDWI is a far simpler algorithm to implement, and has a very similar structure to that proposed (spike-timing based plasticity with an anti-hebbian style). This would be, in my eyes a very simple comparison to add and would complete this work as being truly compared to and contrasted with existing work in this domain. This would be my only remaining request for the work, or at least a convincing explanation as to why not as it has the same kinds of drawbacks as RDD.

(Remarks on code availability)

Version 2:

Reviewer comments:

Reviewer #3

(Remarks to the Author)
Dear Authors,

Thank you very much for your additional effort the updated results. I do believe that this further addition really gives good insight into the differences of your proposal vs STDWI and RDD, and also well motivates why you compared against RDD only in later results.

I find the author changes to have significantly improved the embedding of the work as well as clarity in the results. At this stage I have no further requests and would be in favour of publication of this work.

(Remarks on code availability)

Open Access This Peer Review File is licensed under a Creative Commons Attribution 4.0 International License, which permits use, sharing, adaptation, distribution and reproduction in any medium or format, as long as you give appropriate credit to the original author(s) and the source, provide a link to the Creative Commons license, and indicate if changes were made.
In cases where reviewers are anonymous, credit should be given to 'Anonymous Referee' and the source.
The images or other third party material in this Peer Review File are included in the article's Creative Commons license, unless indicated otherwise in a credit line to the material. If material is not included in the article's Creative Commons license and your intended use is not permitted by statutory regulation or exceeds the permitted use, you will need to obtain permission directly from the copyright holder.
To view a copy of this license, visit https://creativecommons.org/licenses/by/4.0/

Reply to reviewer 1

(1) *Summary: The authors propose a novel method for dynamically symmetrizing connectivity in stochastic spiking networks that learn either via backpropagation or the wake-sleep algorithm for Boltzmann machines. In contrast to Kolen-Pollack learning, which relies on weight decay to symmetrize connectivity, the authors use an anti-causal STDP rule to establish weight symmetry as a line attractor in weight update dynamics (absent other learning signals), as a spiking analog to phaseless alignment learning.*

Overall opinion: The algorithm (SAL) appears novel, and the mathematical proofs supporting it are interesting. The work will likely be of use to researchers in theoretical neuroscience and neuromorphic computing. I think that this paper is worth publishing with the addition of some empirical comparisons to alternative learning algorithms that highlight the algorithmic merit and unique biological implications of SAL.

We were delighted to see your appreciation of our work and thank you for the comprehensive feedback and thorough review. While definitely challenging in their scope, your suggestions helped us shape a much stronger manuscript, for which we are truly grateful. Please find our point-by-point replies below. In summary, beyond multiple smaller changes in response your comments, we have especially added two major new sections to the manuscript, discussing an empirical comparison to a number of other existing spike-based symmetrization mechanisms [1–4], as well as discussing the amenability of spike-based alignment learning (SAL) to networks that obey Dale's law [5].

Before continuing, we find it important to address a side remark in your reply above, namely that SAL establishes weight symmetry in absence of other learning signals. While it is true that SAL can be applied during a dedicated symmetrization phase, this is often not necessary. Indeed, SAL can operate concomitantly with other (functional) learning rules (line 176 ff.), as we also explicitly show in section 2.4.1, where SAL co-occurs with the sleep phase (line 478 ff.). Similarly, as SAL can operate on only one of the reciprocal weights between a pair of neurons (for example the backward weights in functionally feedforward networks, see also line 674 ff., or the EE weights in networks that obey Dale's law, see also line 723 ff.), it can be straightforwardly used in parallel with a different functional learning rule acting on the other one of the two reciprocal weights. This is now explicitly pointed out in the revised discussion on line 1116 ff.

(2) ***Major criticisms:***
1. In recent years, there has been a proliferation of methods that can achieve weight symmetry (or alternative types of learning without weight transport), to the point that one has very little idea of how to compare these algorithms. Many symmetrization methods use only local information, and many are capable of training neural networks on simple datasets (e.g. MNIST, CIFAR-10). The authors do a fairly good job of reviewing these different algorithms in the intro and discussion, but do not provide any concrete empirical comparisons beyond showing superior learning in comparison to feedback alignment, which is by now well known to perform poorly on even relatively simple tasks.

We acknowledge the missing quantitative comparison of SAL against comparable algorithms. Therefore, we have added the new section 2.4.3, in which we assess the performance of SAL and some of its most notable analogs (more specifically, regression discontinuity design (RDD) [2] and Kolen-Pollack algorithm (KP) [3]) using the experimental design proposed in [2].

In summary, we find that SAL enables slightly higher performance (fig. 7c), while producing significantly better alignment than RDD (fig. 7b) and being significantly more robust to noise than KP (fig. 14). Maybe even more importantly, SAL does not need a separate phase of its own, as discussed above. The use of a separate phase in the SymmNet setup is, as discussed on line 636 ff., to isolate the effects of symmetrization from those of functional plasticity, as well as to enable the comparison to these other methods in the first place.

(3) *In particular, I think that the authors have been unfair to the Kolen-Pollack algorithm in several places, and that empirical/mathematical demonstrations are required to support their claims. They introduce a concept called the 'weight update transport problem' in both the introduction and discussion, which argues that the symmetric updates in the Kolen-Pollack algorithm are implausible. However, for Boltzmann machines for instance, the KP algorithm is incredibly simple, amounting to adding a constant weight decay term to the ordinary Hebbian-style update. An argument as to why this type of update is less plausible than the SAL algorithm requires much more explanation in my opinion. Furthermore, in terms of performance, I think that the experiments that the authors conduct are highly unlikely to reveal that SAL is an improvement over KP. This is not to say that SAL doesn't have advantages–I just think that different experimental and/or mathematical demonstrations will likely be required. For instance, the use of a constant decay term in the KP algorithm could present serious issues for continual learning, which the SAL algorithm is unlikely to suffer from (because it establishes symmetric weights as a line attractor).*

We agree with many of your comments, but do not believe to have been unfair to KP. To further substantiate our statements, we have thus gladly accepted your challenge to provide more evidence and better explanations. In particular, we have added results on KP to both the sampling networks in section 2.4.1 (figs. 4 and 5) and the deep hierarchical networks in section 2.4.3 (fig. 7). To anticipate our conclusion: we find SAL to have many advantages over KP, especially in spiking networks.

1. We agree that the constant decay rate of KP indeed amounts the simplest conceivable term in a plasticity rule. That being said, as SAL effectively boils down to a variation on standard spike-timing-dependent plasticity (STDP), we consider it to be quite simple as well, and notably much simpler than other spiking algorithms such as RDD, while straightforwardly compatible with both biological observations and standard neuromorphic implementations of STDP.

2. But more importantly, the weight update transport problem represents a challenging issue in realistic (i.e., noisy) systems – biological and neuromorphic alike – even when the formal functional update rule is symmetric by construction, as you point out for wake-sleep learning in Boltzmann machines. The problem appears when noise affects the functional plasticity mechanism, which is individual to every physical synapse and can thus not be guaranteed to be identical for reciprocal synapses (see our new fig. 5) If weight updates are asymmetric, then a symmetric weight decay cannot correct for the ensuing asymmetry, unless of course it is so strong that it suppresses functional learning itself. This would manifest similarly to the continual learning issue you have rightfully pointed out, only on much shorter time scales.

3. One could argue that this issue can be addressed by optimizing the decay term itself. We have thus studied this possibility in the new section 5.2 and fig. 14. In summary, the optimal decay rate depends on the noise level and would thus need to be adjusted for each system individually, while the tradeoff for larger decay rates in scenarios with larger noise is to the clear detriment of the network performance, as discussed above.

4. That being said, weight decay has its shortcomings even for symmetric weight updates, because it necessarily interacts with functional learning. As networks approach their local performance optimum, functional weight updates naturally become smaller, eventually competing against the KP decay. This phenomenon can be mitigated by smaller decay rates, but this of course results in a weakening of the symmetrization effect itself and poorer functional performance because of the consequently more misaligned weights, not to mention. This explains, for example, the slightly lower performance of KP compared to SAL on all datasets in fig. 7. We discuss this explicitly on line 529 ff. for the sampling networks and on line 671 ff. for the deep networks. Note also that in this setup, some form of weight update transport is needed for adapting the backward synapses, which at least in our model do not attach to the same compartments and thus do not easily have access to the same pre- and postsynaptic information as the forward synapses.
   SAL, in turn, faces none of these problems, because it acts independently on each synapse

and does not interact destructively with the functional learning mechanism (wake-sleep, error backprop etc.).

(4) *2. Related to the issue above, the authors do little to explain how SAL could be validated at an experimental level, relative to alternative symmetrization algorithms. SAL uses only ingredients that have been observed biologically (various types of STDP curves), but so does Kolen-Pollack, for instance, requiring in the case of Boltzmann machines essentially only Hebbian plasticity and gradually decaying synaptic strengths. The authors allude to SAL's ability to better accommodate neurons with differing physiological properties, but do not provide comparisons to alternative symmetrization algorithms. This is a missed opportunity in my opinion–for instance, in neuron models with multiple synaptic connections from neuron A to neuron B, KP and SAL may differ in their predictions for how these multiple synapses should jointly evolve throughout time, and may provide a method to experimentally differentiate between the two algorithms. Providing empirical evidence that SAL is better able to accommodate neural networks with E-I separation would also be beneficial, as this is a claim that the authors make textually but do not empirically substantiate.*

Your comment addresses an essential point, which is also related to the one above, albeit with a more biological touch. Your suggestions are exactly what we had in mind when writing the manuscript, so your request for better motivation was a welcome call to action.

First, we have augmented our demonstration of SAL facing plasticity noise with a study of KP's inability to match SAL's performance in such scenarios (new data in fig. 5 and discussion on lines 552 ff., as well as the new section 5.2 and fig. 14). As discussed above, this includes neuronal diversity as a subcase and clearly showcases an important advantage of SAL with respect to KP. We further note an interesting parallel with a different algorithm (natural gradient descent) that was investigated in spiking networks for very similar reasons, namely the specific desire to address neuronal morphology and its diversity (line 1058 ff.)

Second, we now have an entire section dedicated to Dale's law and the efficacy of SAL in such networks (section 2.5 and fig. 9). As you suggest, this clearly differentiates SAL from rules that must apply to all synapses simultaneously and equally (such as KP), providing strong arguments for its advantages in both biological and artificial networks, while outlining clear differences with respect to experimental predictions.

Altogether, the focus on effective (rather than numerical) weights and SAL's ability to symmetrize while affecting only a subset of weights is now also summarized in the Discussion (line 1183 ff.).

(5) *3. Lastly, the authors do not adequately compare SAL to algorithms that do not require feedback symmetry. They cite and briefly discuss pseudo-inverse-based learning algorithms like difference target propagation and the wake-sleep algorithm for Helmholtz machines, but do not provide any actual comparisons: this is particularly problematic, because the authors do not justify the biological plausibility of symmetric connectivity in the first place. To my knowledge, symmetric connectivity is only justifiable at the level of a very coarse approximation between brain areas, and certainly cannot hold between excitatory and inhibitory neurons (because it violates Dale's law, as the authors themselves discuss). If the weight transport problem can be solved by regression from higher-order cortical areas to lower cortical areas, why should we be worrying about symmetry in feedback connections? In contrast to the KP algorithm, I do not think that empirical comparisons are required here: I would rather see a discussion of the experimental evidence supporting these different families of models.*

We obviously acknowledge the plethora of existing learning algorithms that do not require feedback symmetry, but we have several important reasons for restricting our discussion to those that do (while having extended it substantially, as highlighted in the updated manuscript).

First, we would kindly argue that the sheer volume of literature on symmetry-agnostic cortical learning

algorithms, let alone their experimental correlates, is beyond even the scope of a review paper, and therefore not something we can cover within this manuscript without losing focus (and also considering editorial length limitations). Instead, we would argue – as we do in our Introduction – that an increasing number of very prominent cortical learning models have been put forward that explicitly require (effective) weight symmetry. Our model specifically addresses this class of algorithms and provides an efficient and biologically plausible way to achieve this symmetry.

Second, we would argue that gradient descent algorithms, and in particular error backpropagation, represent some of the most powerful learning paradigms known to date (see lines 1069 ff.). This constitutes the primary reason for the considerable interest in their physical implementation, both in neurobiology and in neuromorphics (see, for example, [6, 7]). All of these models require some form of weight transport, and attempts to sidestep this element (such as feedback alignment (FA) or (difference) target propagation) lack both performance and scalability [8]. We thus believe that SAL closes a considerable gap in such models by proposing a concrete mechanism to either be experimentally validated (in biology) or implemented (in hardware). While efforts in the latter category are currently underway (ongoing work, unpublished), numerous proposals for error representation and transport in cortex have been put forward, many of them implicitly requiring a mechanism such as SAL for scalablility and performance [9–14]. Because existing experimental data is not yet at the level of resolution required to (in)validate these hypotheses, including the required symmetrization mechanisms such as SAL [15–17], recent experimental collaborations have started gathering data for clarifying the microscopic underpinnings of such models [18, 19].

Third, while of course agreeing with your point about detailed symmetry not being compatible with Dale's law, this is not the case for *effective* symmetry, for example between pyramidal cells, which can be connected through multiple pathways, including multapses and also "detours" via inhibitory interneurons. As already discussed above, SAL is explicitly able to instill effective weight symmetry between such neurons. Along with its STDP-like nature, synaptic locality and ability to operate in parallel with other (functional) learning rules, SAL's ability to symmetrize effective weights even when applied to only a subset of synapses constitutes a major argument for its biological plausibility. To our knowledge, none of the other proposed symmetrization algorithms are capable of fulfilling all of these criteria simultaneously, while also achieving the performance of SAL. As already mentioned above in response to your other comments and suggestions, we have added extensive simulations and discussions of these points to the revised manuscript.

(6) ***Minor criticisms:***
1. Line 90: the authors cite the Helmholtz machine 'wake-sleep' learning algorithm, but I believe that they intend to cite the original Boltzmann machine paper (or possibly the contrastive divergence learning algorithm), as this is the method that they actually use. People sometimes refer to the contrastive divergence learning algorithm as a 'wake-sleep' learning algorithm, which is semantically true but liable to cause confusion. The Wake-Sleep learning algorithm for Helmholtz machines does not require feedforward and feedback weight symmetry, which could make a mistaken citation particularly confusing in this context.

Thank you for pointing this out. While "wake-sleep" indeed refers to a different implementation depending on context, the core idea of having two phases in which a network either receives or generates sensory information is what they have in common. Thus, when talking about wake-sleep in the context of Boltzmann machines, we refer to, e.g., the training algorithm in [20], or its spiking variant [21]. Our references were indeed misleading and have been corrected (lines 47 and 1452).

(7) *2.Line 748-9: the claim that mirrored STDP cannot equilibrate initial weight differences is confusing. If it is a variant of the Kolen-Pollack algorithm, which does cause weight symmetry, why would mirrored STDP fail to do this?*

Agreed, the sentence in line 1025 was confusing and we removed it.

(8) *3. Line 999: I do not think that $W_{12} = W_{21}$ is a unique fixed point. The entire argument is that this equality applies to an entire continuous line attractor, not a unique fixed point.*

As we show in section 4.2.2, SAL converges for any starting points W'_{12}, W'_{21} to their common mean $\frac{1}{2}(W'_{12} + W'_{21})$ (when SAL is applied to both weights), which is equivalent to $W_{12} = W_{21}$. Hence all solutions on the line $W_{12} = W_{21}$ are fixed points.

SAL builds on the fundamental property that the spike-timing difference distributions (STDDs) are the same (just mirrored across the y-axis) between two reciprocal synapses. As a direct consequence, the SAL weight updates always[1] obey $\Delta W_{ij} = \Delta W_{ji}$ (see the explanation in lines 352 ff.).

In other words, the trajectory of weights under SAL in the W_{ij}-W_{ji} plane is always strictly *perpendicular* to the diagonal. Hence vanilla SAL always conserves $W = (W_{ij} + W_{ji})/2$ for some initial W_{ij} and W_{ji} and stochastically converges towards $W_{ij} = W_{ji} = W$, which is what we call our fixed point and it is unique for these initial W_{ij} and W_{ji}.

Complementary to SAL, which inherently conserves W, it is the role of any functional learning rule (i.e. backpropagation (BP) or wake-sleep,...) to adjust W in the W_{ij}-W_{ji} space on trajectories *parallel* to the diagonal to reduce an external, task dependent-dependent cost.

(9) *4. Does the proof in section 4.2.2 apply equally well to a full recurrent neural network, or is it fundamentally restricted to the 2 neuron case?*

The proof only makes very basic assumptions about the nature of the neurons in the discussed pair, such as the antisymmetry of the STDP kernel (but not its shape) and the symmetry of the STDD for symmetric effective weights (but again, not its shape). This second assumption might not strictly hold under certain conditions, for example for non-rectangular PSPs (Fig. 10) or if the two neurons receive strongly correlated, asymmetric presynaptic input. However, these effects tend to be small, so while the proof only strictly holds under the assumptions provided explicitly therein, it should also hold approximately in a wide variety of scenarios, as demonstrated in practice in the various setups studied in the paper. Most importantly, the principle still holds, even if the stable fixed points may slightly deviate from the diagonal.

(10) *5. The authors claim to provide the first spiking network implementation of the learning algorithm described in the Sacramento et al. 2018 paper. It would be good to cite and discuss the Guerguiev et al. 2017 paper, "Towards deep learning with segregated dendrites", which does use spiking neural networks in conjunction with a bio-plausible error propagation algorithm.*

Thank you, this is indeed a particularly interesting reference for SAL, and we have added it to the Discussion (line 1051 ff.)

[1] Under ideal conditions, i.e., identical learning rates for both synapses

Reply to reviewer 2

(1) *Summary*

This paper presents a mechanism called spiked-based alignment learning (SAL), allowing to align/symmetrize reciprocal weights based on a particular form of spike-timing dependent plasticity (STDP) arising from the spike-timing difference distribution (STDD) between two neurons. It relies on the presence of noise in neuronal systems to get this STDD information. This paper demonstrates the functionality of SAL in two use cases: (i) a spiking sampling network (SSN) relying on a wake-sleep training and (ii) a dendritic cortical microcircuit learning a nonlinear input-output mapping, with a teacher network training a student one. In addition, this paper shows that SAL remains valid for different types of post-synaptic potentials (PSPs) – rectangular, alpha-shaped – and discusses the implications of different PSPs on the algorithm. One of the key claims of SAL is that is well-suited to analog hardware as it operates based on noise and is robust against components' mismatch.

Thank you for your comprehensive feedback. Please find below a detailed reply to all of your questions and suggestions.

(2) *Strengths and weaknesses*

The main strength of this paper is the idea of using STDD information to enable the alignment of reciprocal weights in neuronal systems, subject to both noise and mismatch. Significant work is provided in demonstrating this mechanism based on simulation results in different conditions (two use cases, different PSPs, etc), and based on theoretical proofs at the end of the paper. This key claim is thus well-supported by the different experiments and results discussed in the current version of the paper.

The main weakness is that the use cases tackled with the proposed SAL technique remain at a relatively low level, in the sense that these are relatively simple problems, and it is not extremely clear to me how this will extend to more challenging problems. I would thus encourage the authors to clarify the computational overhead of SAL and the extent to which noise is necessary for it to work and impacts the learning process. In addition, results on at least one conventional spiking dataset would be of great value to the reader and would provide some intuition about how the proposed SAL technique can scale to more complex problems.

We are delighted to see your appreciation of our theory, and we hope that we were able to address all of your concerns below, in particular regarding the scaling of SAL to deeper architectures and more challenging problems. We have extended our manuscript with a section in which we train a deep convolutional neural network on several challenging datasets (CIFAR-10, Fashion-MNIST, SVHN) with the twin goals of proving SAL's efficiency in such powerful architectures and of conducting a quantitative comparison between SAL and other spiking and non-spiking symmetrization algorithms (see section 2.4.3 and fig. 7).

(3) *Major comments*

• *What is the computational overhead of SAL ? What would be the best way to implement it in an analog substrate ?*

Thank you for this interesting question, this is indeed a central point to examine and discuss, especially when comparing SAL to similar symmetrization algorithms.

Regarding the computational overhead, we carried out a new, large-scale simulation study in section 2.4.3, where SAL was executed in a separate phase with fixed forward weights for different learning rates. This provides two important insights.

First, we see how fast the $\boldsymbol{B}$s and $\boldsymbol{W}$s align in a competitive deep network as a function of time. This provides an estimate for the *maximal* additional time required to symmetrize weights. Note that this is only if SAL is applied in a phased manner, which needs not be the case and was only done here

to disentangle forward from backward learning and allow a direct comparison with other algorithms that require a separate symmetrization phase. *Maximal*, because in real-world cases, when the $\boldsymbol{W}$s aren't fixed, feedback alignment stills happens in the first few epochs and symmetrization happens faster (compare the time scales in fig. 7b and fig. 8). We consider this to be an upper limit because especially during early learning, the naturally occurring alignment of forward to backward weights (i.e., FA) aids symmetrization in the topmost layers. Even so, the overhead imposed by SAL is rather negligible compared to the time required to learn the forward weights, as discussed on line 686 ff.

Second, fig. 8 shows how alignment angles can be further improved by choosing smaller SAL learning rates; note that this only affects total training times if symmetrization phases are separate from forward learning phases (which is not necessary for SAL) or, for simultaneous B-learning, if SAL convergence times are significantly longer than the duration of individual training epochs (which is not the case for any of the parameters used in these simulations).

Regarding the implementation in analog substrates, we point to line 1101 ff. of the Discussion. Many neuromorphic architectures already come equipped with various STDP mechanisms, thereby providing the core component of SAL. A straightforward analog implementation of exponential STDP windows uses a single capacitor for each branch. The causal capacitor is charged by presynaptic spikes and read out by postsynaptic spikes, and vice-versa for the anticausal one; the readout value can then be translated into a synaptic update. The other component required by SAL is noise, which is also readily available especially in analog substrates. Depending on the particular design, thermal noise can drive stochasticity, especially in memristive or photonic systems, as discussed for example in [22]. However, explicit sources of noise might not even be necessary, as we discuss in response to your next point below. These minimal requirements also set our approach apart from other spiking symmetrization algorithms such as RDD [2], where additional neuronal variables are required that might be difficult to reconcile with existing platforms, as now discussed in the new version in line 993.

(4) • *One of the key assumptions for SAL to work is that noise is present in the considered system. Therefore, I am wondering how SAL would work in hardware implementations for which intrinsic noise is very limited, for example a digital hardware implementation ? Would SAL still work in that context, or does that depend on the level of noise in the input data ? In addition, is there a minimum level of noise necessary to ensure a proper operation ? How does the noise level impact the convergence speed of the weight alignment ?*

It is true that SAL requires a certain amount of noise to function. This is because neurons need to measurably influence each other's output even for small or inhibitory weights – otherwise a presynaptic spike might never trigger a postsynaptic one, preventing synapses from "measuring" their reciprocals by means of the STDD Mere noise on the input is not enough as it would only affect the input layer.

Mathematically speaking, if the noise is assumed to be Gaussian, any level of noise will suffice, because firing probabilities will then always be nonzero. In practice however, too low noise might slow down convergence, as neurons would need more time to sample often enough from their STDD for SAL to properly take effect. If noise is generated externally, a safe way of setting a sufficiently high noise level is for neurons to fire at about half their maximum firing rate in absence of synaptic input, but much lower levels of external noise are often also sufficient, as discussed further below. We have added a couple of sentences in line 1127 ff. For a more ample exposition of how noise levels affect sampling, we refer to [23].

This noise itself can come from a large variety of sources. As discussed above, it is readily available (and even difficult to evade) in many analog systems, including biological ones. In digital systems, individual neurons can be made noisy very efficiently using pseudorandom number generators such as linear feedback shift registers.

Most importantly however, noise may come in the form of diffuse spiking input, in biology often assumed as (roughly) Poisson-distributed. This does not mean that each neuron needs to have its own

private Poisson source. As was shown in [24], relatively small deterministic networks can provide even large functional networks with pseudo-noise of sufficient quality to allow their unimpeded operation. Moreover, ensembles of functional networks can provide each other with pseudo-noise, without the need for any additional resources invested into the production of noise alone ([25]). At its most extreme, this even allows recurrent networks to feed themselves with noise, i.e., to produce activity that is stochastic enough for neurons to fire as if driven by strong Gaussian noise (Video S3 in [25]).

(5) *• Would that make sense to use some alignment metric to assess the alignment of forward and backward weights, similar to the angle typically used in works related to feedback alignment (FA) ?*

Yes, absolutely. We used it in the new figs. 7 and 8 as *the* metric to compare the alignment. On this note, we would like to point out a detail that might otherwise be overlooked. Although the angle is a very intuitive measure for the weight alignment, it doesn't take into account the magnitude of the weights (i.e. the vector length), which SAL also aligns (fig. 2e).

(6) *• The paper does a good job at demonstrating the operation of SAL but remains limited to some simple problems. I believe the paper would be strengthened if you could demonstrate the proper operation of SAL on at least one conventional yet simple spiking classification dataset, with alignment metrics at the level of whole matrices, if feasible in the time allowed by the review process*

This is a very valid point and has also been raised by Reviewer 3 (Point 7). We have thus put considerable effort into addressing it, which is also the main reason for the duration of the revision. We have now included an entire section with substantial new results (section 2.4.3 with fig. 7), with the two-fold goal to a) convincingly prove SAL's efficiency in competitive deep architectures and b) to systematically compare SAL with other (spiking and non-spiking) symmetrization schemes. This is also why we chose standard, but more difficult datasets as opposed to ones specifically constructed for spiking platforms, as purely spiking datasets might be perceived as diminishing the scope of SAL.

To this end, we followed the approach used in [2], where the forward weights are trained in an artificial neural network (ANN) by standard autograd methods and then transferred to an equivalent spiking network to learn the backward weights with either spiking or non-spiking approaches. This allows a direct comparison with the gold standard in ANNs (BP) for challenging datasets (CIFAR-10, Fashion-MNIST, SVHN), as it disentangles symmetrization from the learning of forward weights. In summary, this approach allowed us to demonstrate distinct advantages of SAL in terms of performance and efficiency compared to other symmetrization approaches. See also our reply to Reviewer 1, Points 2 and 3 for a more detailed discussion of this comparison.

(7) ***Minor comments***
• p.4 Fig. 2: For Fig. 2c1, I would explicitly add that the top graph is for two excitatory weights and the bottom one for inhibitory weights. We can infer that from the weights values on the right side, but I still believe it would be clearer to write excitatory and inhibitory in plain text.

Nice idea. Done.

(8) *For Fig. 2d, I would add a legend for the colors instead of just describing it in the caption.*

Good idea. Done.

(9) *In addition, how does the convergence speed compare with τ_{ref} ? Is there a link between the convergence speed and τ_{ref} ?*

This is a very interesting question, because it touches on the statistical core of SAL, which we also addressed in our reply to your Point 4 above. In general, τ_{ref} controls the maximum firing rate of neurons and thereby also their sampling speed. Because SAL builds on a good enough sampling of the STDD, longer τ_{ref} would correspondingly reduce the convergence speed. This is why we often think of

time in units of τ_{ref}rather than seconds.

In order to allow a quick assessment of the correspondence between these units, we added a second abscissa to fig. 2d. However, it is important to note that this simulation was not optimized for convergence speed. For a more in-depth discussion of convergence in the context of deep learning and more challenging tasks, we refer to the new fig. 8 and associated text (section 2.2). In summary, we observe that the learning rate represents the main control knob for SAL's convergence speed, which comes at a trade-off with alignment precision. In other words, arbitrarily small alignment angles can be reached for appropriately small learning rates, but then also correspondingly long convergence times. That being said, for the performances achieved in fig. 7, SAL convergence times were negligible compared to the times required by forward weight learning, which speaks to the efficiency of our model.

(10) • *p.7 l.538: It should be Fig. 6b2 instead of Fig. 6a2, no ?*

Good catch, thanks.

(11) • *p.8 Fig. 6b2: I would indicate with a vertical line the point at which the B_{12} switches sign and thus provides a meaningful error signal*

Nice idea. Done.

(12) • *p.9 Fig. 7b: I recommend to zoom on the STDD depicted here as it is difficult to see the differences due to the biases based on the current figure*

Dr. Martin Lefebvre, TU Delft

You are right, the differences are subtle, but we are afraid that additional panels would further complicate what already is a rather complicated figure. We have now replotted the figure with larger biases, thus allowing a better differentiation between the lines. We hope that these are now more discernible without the need for an additional zoom-in.

Reply to reviewer 3

(1) *This submitted work is an interesting approach which bridges between spiking (sampling) network modelling, addressing of the weight-transpose problem of credit assignment, and giving a purpose to noise within neural networks. In general it is extremely well written, the figures are clear and cover every desirable aspect of network analysis, and the theory is sound. The overall framing of the problem is also done very well and I enjoyed the perspective.*

Thank you for your kind words! We are delighted that we could spark your interest in our work and that you value the effort we put in writing the manuscript.

(2) *However, there are two major flaws at present which make it difficult to recommend for acceptance. One, a lack of acknowledgement and embedding of this work within a number of papers which already attempt weight alignment in deep and spiking neural networks via noise for parameter estimation (or update alignment). And two, the lack of a test of this method in training of more difficult tasks, task scales at which these methods really could be shown to break down.*

We appreciate this feedback and have therefore invested substantial effort in addressing these shortcomings. In particular, we have added a large-scale simulation study of several symmetrization methods in the new section 2.4.3, as well as a summary of our conclusions and further contextualization in the Discussion (line 993 ff.). We address these in more detail in response to your comments below and hope that they will convince you of the strength and advantages of SAL.

(3) *Returning to the first concern, there is an unfortunate and worrying lack of embedding of this work within the larger literature on this topic. This work, as it is presented, misses out on key papers which have already covered a number of the topics that are attempted herein. Most importantly, two key papers have already considered how in spiking neural networks it might be possible to use spike timing (with or without membrane voltages) in order to estimate symmetric reciprocal weight values. The first, "Spike-based causal inference for weight alignment" by Guerguiev et al. (2019) describes how causal inference (via regression discontinuity design) can be used to directly infer the values of synaptic weights of a forward-driven model for use in a backward pass. Even more similarly to this work, "Overcoming the Weight Transport Problem via Spike-Timing-Dependent Weight Inference" by Ahmad et al. (2020) proposes that an STDP rule may be used with for weight inference that has a similar form to that proposed in this work. Namely an anti-hebbian form which can be shown to carry out a weight inference process of forward weights using spike-timing alone.*

Thank you for pointing out these very interesting references. We carefully examined both approaches and address them in detail in the revised Discussion (line 984 ff.). Additionally, we have performed a qualitative comparison with RDD (see the new section 2.4.3 and fig. 7) in a deep ConvNet architecture trained on more difficult datasets, which also addresses your second concern and is discussed in more detail in the corresponding reply below (Point 7).

In summary, both RDD and spike-timing-dependent weight inference (STDWI) are interesting and relevant approaches to the weight transport problem, but SAL has the distinct advantage of being the much "simpler" model and thus coming with fewer constraints, variables, and assumptions. For instance, both RDD and STDWI are applied together with a delicate stimulation protocol, while SAL can run concurrently to the functional training, as shown in the sampling experiments in section 2.4.1. But even if SAL was to be implemented in a separate phase, then it remains a much simpler undertaking, because the whole network is put into a global "symmetrization mode" in which all synapses are aligned concurrently, as opposed to RDD and STDWI, where subgroups of neurons need to be stimulated sequentially. Another aspect concerns the model complexity: SAL only requires STDP and noise, while RDD comes with a significant overhead of new dynamical variables such as the *input drive* or the post-synaptic spike responses, that need to be stored by some physical mechanism in the

neurons, or a per-synapse fitting mechanism for the piecewise linear model.

Similarly, STDWI uses *pseudo-synapses* in the backward pathway, which aren't obviously compatible with our current understanding of biology or existing neuromorphic designs. Altogether, while we obviously cannot rule out that simple and efficient solutions to these implementation problems exist, the papers do not provide implementation details for either artificial or biological neuronal networks.

(4) *Aside from these two (extremely relevant) works which aught to be considered, a paper by Akrout et al. 2020 ("Deep Learning without Weight Transport") is not cited and is close to a rate-equivalent alternative to SAL (better to compare against that the Kolen-Pollack algorithm.*

Agreed, and thank you for pointing us to this reference. We now discuss [3] in detail, both in the new simulation results (section 2.4.3) and the discussion (line 936 ff.). We further note that [3] actually contains two different algorithms:

Section 5 in the paper describes a version of the KP algorithm. This is the one we refer and compare to to in our manuscript as in fig. 7.

Section 4, on the other hand, describes the weight mirror (WM) algorithm (lines 955 ff.). This shares some similarity with SAL, and especially with its rate-based sibling PAL, as you correctly remark, because it uses noise for weight transport. The ability of SAL to operate in spiking networks thus constitutes the most important difference, but there are other major differences still. For example, WM assumes linear activation functions for perfect weight transport, which SAL manifestly does not. Also, WM requires neurons to be driven purely by independent, i.e., uncorrelated noise, which SAL also does not; for example, in the sampling networks in section 2.4.1, any pair of neurons may be highly correlated simply by sharing multiple (stochastic) presynaptic partners, as long as each neuron receives enough uncorrelated noise. Furthermore, WM is proposed in the context of a very specific circuit, with strong lateral projections that appear between the pathways during the mirroring phase and then disappear again, and with ideally sequential activations of consecutive layers in order to avoid shared-input correlations. None of these are necessary for SAL. At the same time, WM seems to behave very similarly to KP in the simulations performed by the authors, and we do compare SAL with KP in the new section 2.4.3.

(5) *Further, "Biologically-plausible learning algorithms can scale to large datasets" by Xiao et al. (2018) provides more large scale evidence for sign-alignment as a fruitful direction.*

We already discussed sign-alignment in the old manuscript (see line 926) and showed that SAL can do significantly better – namely actual alignment – without adding much complexity. This is essential for the sampling networks in section 2.4.1, where mere sign-alignment would be insufficient for reasonable performance. Nontheless, while omitting it from the new fig. 7 to avoid clutter, we also compare SAL to a sign-concordant FA algorithm in table 5.

In this context, we should also note that, despite their apparent simplicity, sign-concordant FA algorithms come with their own challenges. Not only do they require a mechanism for sign transport, but they also face tensions with biology when considering Dale's law, on which we also included the new section section 2.5.

(6) *These concerns around embedding within the existing literature are significant from my perspective. Their exclusion (especially Guerguiev et al. and Ahmad et al.) means that this work is not compared against true contemporary and comparable research. The existence of these works also impacts the novelty of this approach.*

We took your critique to heart and hope to have now alleviated your concerns. Given our now thorough comparison with these alternative models, we believe to have significantly strengthened the arguments for SAL's simplicity, biological plausibility, neuromorphic amenability, resource efficiency and performance.

(7) *My second major concern lies around the scale of tasks attempted by this work. Though I acknowledge that machine learning benchmarks are not the only important tasks, all serious breakdowns of training by FA/DFA/TP or other 'biologically-plausible' algorithms tend to breakdown only at a certain scale of task complexity (specifically close to ImageNet if one considers the work by Bartunov et al. 2018). Given this, I would be curious to see how well SAL truly does when applied in networks to attempting such tasks (though I also understand that this require a significant network change).*

We clearly see how scaling to more complex tasks (and corresponding network architectures) would strengthen our manuscript, which is why we did just that. In our new section 2.4.3, we compare SAL with several of its competitors in convolutional networks trained on CIFAR-10, Fashion-MNIST and SVHN. For this, we use the setup from one of the references you suggested [2], which they used to assess the performance of their own weight tranport algorithm (RDD). In summary, we find SAL to compare favorably with the other models in terms of pure network performance. Furthermore, we note that we have chosen relatively large SAL learning rates in order to keep convergence times short. As shown in fig. 8, if given small enough learning rates and long enough time, SAL can achieve arbitrarily small alignment angles and thus enable performance arbitrarily close to vanilla BP. For more details, beyond these additions to the manuscript, we would also point to our replies to Reviewer 2, Point 6, and Reviewer 1, Points 2 and 3.

(8) *One final small concern is that it appears that Figure 5b1 shows that the divergence begins to grow near the end of training (as does the variance across models) for the SAL model. Is this indeed the case? Would these networks diverge in accuracy if allowed to train for longer? If so this should be a concern, so I'd like to see longer simulation time for these plots.*

This was an excellent catch that in turn allowed us to catch a bug in our code, thank you! This has now been fixed in the new version (see fig. 5). Here's the explanation: What the code was supposed to do was $\Delta W_{ij}^{\text{wake}} = \eta(\alpha_{ij} f(\Delta t_{ij}))$ and $\Delta W_{ij}^{\text{sleep}} = \eta(-\alpha_{ij} f(\Delta t))$, where a synapse-specific noise factor α_{ij} is applied. However, the α was not correctly applied to the wake phase, resulting in a fundamental imbalance between the wake and sleep states that distorted the wake-sleep algorithm and had nothing to do with SAL. In the new version, α is applied as intended and the divergence is gone.

(9) *Overall, I think there are a number of aspects of this work that are truly interesting and novel, including the solving of weight-transport within models with simultaneously active forward and backward weights, the clear theoretical relationship to spike timing differences, and the strong convergence properties and demonstrations. It is also extremely well written, formatted, and presented. However in it's current state this paper sidelines much of existing work that has been done in this direction and this should be rectified.*

We thank you again for your kind assessment of our paper's strengths and for your important inputs. We believe to have crafted a much stronger paper in the revision process and hope to have fully addressed all of your concerns.

References

1. Ahmad, N., Ambrogioni, L. & van Gerven, M. A. Overcoming the weight transport problem via spike-timing-dependent weight inference. *arXiv preprint arXiv:2003.03988* (2020).
2. Guerguiev, J., Kording, K. P. & Richards, B. A. Spike-Based Causal Inference for Weight Alignment. *arXiv:1910.01689.* arXiv: `1910.01689` (2019).
3. Akrout, M., Wilson, C., Humphreys, P., Lillicrap, T. & Tweed, D. B. *Deep Learning without Weight Transport* in *Advances in Neural Information Processing Systems* **32** (Curran Associates, Inc., 2019).
4. Max, K. *et al.* Learning Efficient Backprojections across Cortical Hierarchies in Real Time. *Nature Machine Intelligence* **6,** 619–630. ISSN: 2522-5839 (June 2024).

5. Dale, H. Pharmacology and Nerve-Endings. *Proceedings of the Royal Society of Medicine* **28,** 319–332. ISSN: 0035-9157. http://dx.doi.org/10.1177/003591573502800330 (Jan. 1935).
6. Richards, B. A. *et al.* A Deep Learning Framework for Neuroscience. *Nature Neuroscience* **22,** 1761–1770. ISSN: 1546-1726 (Nov. 2019).
7. Pfeiffer, M. & Pfeil, T. Deep Learning With Spiking Neurons: Opportunities and Challenges. *Frontiers in Neuroscience* **12.** ISSN: 1662-453X. http://dx.doi.org/10.3389/fnins.2018.00774 (Oct. 2018).
8. Bartunov, S. *et al.* *Assessing the Scalability of Biologically-Motivated Deep Learning Algorithms and Architectures* in *Advances in Neural Information Processing Systems* **31** (Curran Associates, Inc., 2018). https://proceedings.neurips.cc/paper/2018/hash/63c3ddcc7b23daa1e42dc41f9a44a873-Abstract.html.
9. Whittington, J. C. R. & Bogacz, R. An Approximation of the Error Backpropagation Algorithm in a Predictive Coding Network with Local Hebbian Synaptic Plasticity. *Neural Computation* **29,** 1229–1262. ISSN: 0899-7667 (May 2017).
10. Guerguiev, J., Lillicrap, T. P. & Richards, B. A. Towards Deep Learning with Segregated Dendrites. *eLife* **6** (ed Latham, P.) e22901. ISSN: 2050-084X (Dec. 2017).
11. Sacramento, J., Ponte Costa, R., Bengio, Y. & Senn, W. *Dendritic Cortical Microcircuits Approximate the Backpropagation Algorithm* in *Advances in Neural Information Processing Systems* (eds Bengio, S. *et al.*) **31** (Curran Associates, Inc., 2018). https://proceedings.neurips.cc/paper_files/paper/2018/file/1dc3a89d0d440ba31729b0ba74b93a33-Paper.pdf.
12. Payeur, A., Guerguiev, J., Zenke, F., Richards, B. A. & Naud, R. Burst-Dependent Synaptic Plasticity Can Coordinate Learning in Hierarchical Circuits. *Nature Neuroscience* **24,** 1010–1019. ISSN: 1546-1726 (July 2021).
13. Mikulasch, F. A., Rudelt, L., Wibral, M. & Priesemann, V. Where is the error? Hierarchical predictive coding through dendritic error computation. *Trends in Neurosciences* **46,** 45–59 (2023).
14. Max, K., Jaras, I., Granier, A., Wilmes, K. A. & Petrovici, M. A. 'Backpropagation and the brain'realized in cortical error neuron microcircuits. *bioRxiv,* 2025–07 (2025).
15. Jordan, R. & Keller, G. B. Opposing influence of top-down and bottom-up input on excitatory layer 2/3 neurons in mouse primary visual cortex. *Neuron* **108,** 1194–1206 (2020).
16. O'Toole, S. M., Oyibo, H. K. & Keller, G. B. Molecularly Targetable Cell Types in Mouse Visual Cortex Have Distinguishable Prediction Error Responses. *Neuron* **111,** 2918–2928.e8. ISSN: 0896-6273 (Sept. 2023).
17. Audette, N. J. & Schneider, D. M. Stimulus-specific prediction error neurons in mouse auditory cortex. *Journal of Neuroscience* **43,** 7119–7129 (2023).
18. Aizenbud, I. *et al.* Neural mechanisms of predictive processing: a collaborative community experiment through the OpenScope program. arXiv. *arXiv preprint arXiv:2504.09614* (2025).
19. The MICrONS Consortium *et al.* Functional Connectomics Spanning Multiple Areas of Mouse Visual Cortex. en. *Nature* **640,** 435–447. ISSN: 1476-4687. https://www.nature.com/articles/s41586-025-08790-w (Apr. 2025).
20. Ackley, D. H., Hinton, G. E. & Sejnowski, T. J. A Learning Algorithm for Boltzmann Machines*. *Cognitive Science* **9,** 147–169. ISSN: 0364-0213, 1551-6709 (Jan. 1985).
21. Neftci, E., Das, S., Pedroni, B., Kreutz-Delgado, K. & Cauwenberghs, G. Event-driven contrastive divergence for spiking neuromorphic systems. *Frontiers in neuroscience* **7,** 272 (2014).
22. Boikov, I. K., de Rossi, A. & Petrovici, M. A. Ultrafast neural sampling with spiking nanolasers. *Nature Communications.* ISSN: 2041-1723. http://dx.doi.org/10.1038/s41467-025-66818-1 (Dec. 2025).
23. Korcsak-Gorzo, A. *et al.* Cortical Oscillations Support Sampling-Based Computations in Spiking Neural Networks. *PLOS Computational Biology* **18,** e1009753. ISSN: 1553-7358 (Mar. 2022).

24. Jordan, J. *et al.* Deterministic networks for probabilistic computing. *Scientific reports* **9,** 18303 (2019).

25. Dold, D. *et al.* Stochasticity from Function — Why the Bayesian Brain May Need No Noise. *Neural Networks* **119,** 200–213. ISSN: 0893-6080 (Nov. 2019).

Reply to reviewer 2

(1) *I thank the authors for their work in addressing my comments. With the added results on common machine learning datasets, as well as with the comparison to other conventional feedback alignment algorithms, I believe the paper is now ready for publication and do not have any further comments.*

Dr. Martin Lefebvre, TU Delft

Thank you again for your constructive feedback and encouraging comments.

Reply to reviewer 3

(1) *I want to start by commending the authors on significant improvements and integrations of this work with existing literature in the new revision. It is much improved and addresses almost all of my concerns.*

We are very happy to see your appreciation, thank you!

(2) *The added comparison to RDD is a great addition and likely involved a lot of work. However, I find it odd that the authors chose not to also compare against STDWI? STDWI is a far simpler algorithm to implement, and has a very similar structure to that proposed (spike-timing based plasticity with an anti-hebbian style). This would be, in my eyes a very simple comparison to add and would complete this work as being truly compared to and contrasted with existing work in this domain. This would be my only remaining request for the work, or at least a convincing explanation as to why not as it has the same kinds of drawbacks as RDD.*

Thank you for pointing out the missing comparison with spike-timing-dependent weight inference (STDWI). The omission of a specific comparison with STDWI in the previous version was intentional. Our considerations were as follows:

Pseudo backward connections The STDWI algorithm is built around the rather unusual assumption of *pseudo* backward synapses, i.e., synapses that have no effect on their postsynaptic neuron but can still be updated by spike-timing-dependent plasticity (STDP). To us, this design choice undermines the model's biological plausibility. More importantly however, this has a very critical algorithmic implication: The weight updates of the STDWI learning rule depend exclusively on the forward weights and not the backward weights. The lack of feedback from (potentially converging) feedback weights means that the STDWI algorithm doesn't know it should "stop" upon reaching symmetry. Instead, divergence is prevented by a weight decay term in equation 9 in [1] that cannot, in general, be $\boldsymbol{W}$-specific. In contrast, spike-based alignment learning (SAL) is able to ensure convergence towards $\boldsymbol{B} = \boldsymbol{W}$ (and not just convergence of the sign, or convergence to some proportional value) precisely because both synapses are active simultaneously, allowing the spike-timing difference distribution to reflect the true weight asymmetry.

Weight units In the SymmNet framework, weights have to be transferred between an artificial neural network (ANN) and a spiking network, which requires to convert the units of the weights. As discussed above, the scale (or unit) of the (converged) feedback weights in STDWI depends on the choice of hyperparameters (such as α or η). Therefore, it is unclear how to convert the aligned $\boldsymbol{B}$s back to the ANN domain.

Integration in deeper models The STDWI does not include simulations with more than two layers. This is particular problematic for the SymmNet framework, because it is unclear how STDWI behaves in such more realistic settings, especially when the upper layer also receives activity from other sources. In contrast to that, SAL symmetrizes the feedback connections in all layers at the same time and is therefore compatible with arbitrary network depths.

Nonetheless, we agree that a quantitative comparison of STDWI would help highlight the advantages of our method. Given that the original STDWI code was not designed for deeper networks, substantial work would have been required to make it fit for the deep SymmNet simulations. Instead, we ran simulations of two-layer networks, in an attempt to get as close as possible to the scenarios studied in [1], such as their Fig. 4. In our new fig. 7, we compare the convergence of all three spiking symmetrization algorithms – SAL, regression discontinuity design (RDD) and STDWI – in a network that mimics the two layers of the deep SymmNet setup.

We hope that these new results further strengthen our paper and directly answer your remaining

request.

References

1. Ahmad, N., Ambrogioni, L. & van Gerven, M. A. Overcoming the weight transport problem via spike-timing-dependent weight inference. *arXiv preprint arXiv:2003.03988* (2020).
